# Supplementary material for: The coevolution of the firm and the product attribute space
Source: PLoS One. 2020 Jun 12;15(6):e0234007. doi: 10.1371/journal.pone.0234007 (PMC7292365; doi:10.1371/journal.pone.0234007)
Supplement: S1 File — (DOCX) [file pone.0234007.s001.docx]

**S1 File**

**Supplementary Information**

**A. Mathematical derivation of the multi-variant Cournot model.**

Each firm *i* calculates its profit from variant *j* according to:

$\pi_{i,j}={P_{j}q}_{i}-c_{r,j}q_{i,j}$ , (SP1)

with $P_{j}$ being the price at variant *j*, $q_{i,j}$ the quantity firm *i* produces at *j*, $q_{i}$ the total quantity offered at *j*, $c_{r,j}$ the unit production cost, and *r* the firm type (*α* or *β*). A population of $n_{j}$ firms offers product variant *j*, of which $n_{\alpha,j}$ are *α-type* and $n_{\alpha,j}$ are *β-type*. The price equation of *j* is defined as:

$P_{j}=a-b_{j}Q_{j}=a-b_{j}\sum_{i=1}^{n_{j}} q_{i,j}$ , (SP2)

Instantiating Equation (SP2) in Equation (SP1), a given firm *i* that takes advantage of scale economies (i.e., *α-type*) maximizes its benefits at variant *j* when:

$\frac{\partial\pi_{i,j}}{\partial q_{i,j}}=a-b_{j}\sum_{z=1, z\neq i}^{n_{j}} q_{z,j}-2b_{j}q_{i,j}-c_{,j}+2e_{j}q_{i,j}=0, i=1,2,\ldots n_{\alpha,j}$, (SP3)

and

$\frac{\partial\pi_{i,j}}{\partial q_{i,j}}=a-b_{j}Q_{j}+q_{i,j}\left( 2e_{j}-b_{j} \right)-c_{,j}=0, i=1,2,\ldots n_{\alpha,j}$. (SP4)

Summing up over all $n_{\alpha,j}$ firms, we get:

$n_{\alpha,j}a-n_{\alpha,j}b_{j}Q_{j}+(2e_{j}-b_{j})\sum_{i=1}^{n_{\alpha,j}} q_{i,j}-n_{\alpha,j}c_{,j}=0$. (SP5)

In an analogous way, for a *β-type* firm, we obtain:

$$\frac{\partial\pi_{i,j}}{\partial q_{i,j}}=a-b_{j}\sum_{z=1, z\neq i}^{n_{j}} q_{z,j}-2{b_{j}q}_{i,j}-c_{,j}=0,$$

$i=n_{\alpha,j}+1,n_{\alpha,j}+2,\ldots,n_{\alpha,j}+n_{\beta,j}$. (SP6)

Thus, summing up over all $n_{\beta,j}$ firms, we have:

$n_{\beta,j}a-n_{\beta,j}b_{j}Q_{j}-b_{j}\sum_{i=n_{\alpha,j}+1}^{n_{\alpha,j}+n_{\beta,j}} q_{i,j}-n_{\beta,j}c_{\beta,j}=0$. (SP7)

Knowing that the total number of firms in variant *j* is $n_{j}=n_{\alpha,j}+n_{\beta,j}$, and that $Q_{j}=\sum_{i=1}^{n_{\alpha,j}} q_{i,j}+ \sum_{i=n_{\alpha,j}+1}^{n_{\alpha,j}+n_{\beta,j}} q_{i,j}$, we proceed to multiply Equation (SP7) by $-(2e_{j}-b_{j})/b_{j}$ and add it to Equation (SP5). Then we get:

$\left[ n_{\alpha,j}-[\frac{\left( 2e_{j}-b_{j} \right)}{b_{j}}]n_{\beta,j} \right]a+\left[ n_{\beta,j}\left( 2e_{j}-b_{j} \right)-{b_{j}n}_{\alpha,j} \right]Q_{j}+\left( 2e_{j}-b_{j} \right)Q_{j}-\left[ n_{\alpha,j}-[\frac{\left( 2e_{j}-b_{j} \right)}{b_{j}}]n_{\beta,j} \right]c_{\beta,j}=0$ , (SP8)

which yields a total quantity at variant *j* of:

$Q_{j}=\frac{n_{\alpha,j}-[\frac{(2e_{j}-b_{j})}{b_{j}}]n_{\beta,j}}{{b_{j}n}_{\alpha,j}-\left( 2e_{j}-b_{j} \right)(n_{\beta,j}+1)}(a-c_{\beta,j})$. (SP9)

Going back to Equations (SP5) and (SP7), and defining $Q_{,j}= \sum_{i=1}^{n_{\alpha,j}} q_{i,j}$ , $Q_{\beta,j}= \sum_{i=n_{\alpha,j}+1}^{n_{\alpha,j}+n_{\beta,j}} q_{i,j}$, we get:

$Q_{,j}=\frac{n_{\alpha,j}(a-{b_{j}Q}_{\beta,j}-c_{\beta,j})}{b_{j}n_{\alpha,j}-(2e_{j}-b_{j})}$ , (SP10)

and

$Q_{\beta,j}=\frac{n_{\beta,j}(a-{b_{j}Q}_{,j}-c_{\beta,j})}{b_{j}n_{\beta,j}+b_{j}}$ . (SP11)

Solving by substitution, Equations (SP10) and (SP11) become:

$Q_{,j}=\frac{n_{\alpha,j}}{b_{j}\left( n_{\alpha,j}+n_{\beta,j}+1 \right)-2e_{j}(n_{\beta,j}+1)}(a- c_{\beta,j} )$ , (SP12)

and

$Q_{\beta,j}=\frac{[\frac{(b_{j}-2e_{j})}{b_{j}}]n_{\beta,j}}{b_{j}\left( n_{\alpha,j}+n_{\beta,j}+1 \right)-2e_{j}(n_{\beta,j}+1)}(a- c_{\beta,j} )$ . (SP13)

Then, if firm *i* is *α-type*, its production level at variant *j* would be $q_{i,j}=Q_{\alpha,j}/n_{\alpha,j}$; if it is *β-type*, then its production level would be $q_{i,j}=Q_{\beta,j}/n_{\beta,j}$.

**B. Information about variable / parameter definitions and experimental design**

**Table SP1**. Variable and parameter definitions

| **Denotation** | **Explanation** | **Range of values** |
| --- | --- | --- |
| *M* | Total number of consumer units | 5000 |
| *F* | Total number of firms | [10, 20] |
| $c_{0}$ | Minimum attainable unit cost | 0 |
| $c_{\alpha,j,t}$ | *α*-type firms’ unit production cost | Variable |
| $c_{\beta,j}$ | *β*-type firms’ unit production cost | Uniformly distributed in range [$a$, $e_{j}M$] |
| $e_{j}$ | Unit cost reduction impact per unit of production quantity at variant *j* | Uniformly distributed in range [$0.45$, $0.50$) |
| $\pi_{i,j}$ | Profit of firm *i* at variant *j* | Variable |
| $P_{j}$ | Price of variant *j* | Variable |
| $q_{i,j,t}$ | Firm *i*’s production quantity at variant *j* at time *t* | Variable |
| $q_{i,t}$ | Total firm *i*’s production quantity time *t* | Variable |
| *a* | Price equation intercept | *M* |
| *b_j,t_* | Price equation slope | (0,1] |
| *n_j_* | Total number of firms at variant *j* | Variable |
| *Q_j_* | Total quantity at variant *j* | Variable |
| $n_{\alpha,j,t}$ | Number of *α*-type firms at variant *j* at time *t* | Variable |
| $n_{\beta,j,t}$ | Number of *β*-type firms at variant *j* at time *t* | Variable |
| *N* | Fixed cost of attending a variant (niche spanning) | [500, 1500] |
| *H_i,t_* | Firm *i*’s niche at time *t* | Variable |
| $q_{i,t}$ | Firm *i*’s total production quantity at time *t* | Variable |
| ${\tilde{\pi}'}_{i,t+1}$ | Firm *i*’s profit expectation at time *t*+1 | Variable |
| $\tilde{\pi}_{i,j,t+1}$ | Firm *i*’s profit expectation at variant *j* at time *t*+1 | Variable |
| $s_{H_{i,t}}$ | Largest block distance between any two variants in firm *i*’s niche at time *t*, *H_i,t_* | Variable |
| ${\tilde{\pi}''}_{i,t+1}$ | Firm *i*’s profit expectation at time *t*+1 after inclusion of a new variant | Variable |
| $V_{t}$ | Number of active variants at time *t* | Variable |
| $\tilde{\pi}_{p}$ | Firm *i*’s profit expected monopolistic profits at new variant | Variable |
| ${\tilde{\pi}'''}_{i,t+1}$ | Firm *i*’s profit expectation at time *t*+1 after expanding to an existing variant | Variable |
| ${\tilde{\pi}''''}_{i,t+1}$ | Firm *i*’s profit expectation at time *t*+1 after a variant has been dropped | Variable |
| *d* | Dropped or additional variant | Variable |
| *K* | One-time cost of opening up a new variant | [10000, 25000] |

**Table SP2.** Parameter value scenarios obtained by the Latin Hypercube Sampling procedure for the three variables of interest (number of firms, niche-width cost, and variant opening cost)

| **Scenario** | **Total number of firms (*F*)** | **Niche-width cost (*N*)** | **Variant opening cost (*K*)** |
| --- | --- | --- | --- |
| 1 | 17 | 525 | 21625 |
| 2 | 16 | 1125 | 17875 |
| 3 | 10 | 575 | 15625 |
| 4 | 11 | 775 | 23875 |
| 5 | 12 | 1325 | 20125 |
| 6 | 15 | 1275 | 20875 |
| 7 | 13 | 1025 | 14875 |
| 8 | 11 | 925 | 22375 |
| 9 | 16 | 625 | 14125 |
| 10 | 20 | 725 | 24625 |
| 11 | 18 | 1425 | 23125 |
| 12 | 19 | 825 | 12625 |
| 13 | 14 | 1475 | 13375 |
| 14 | 14 | 1375 | 16375 |
| 15 | 19 | 875 | 17125 |
| 16 | 17 | 1175 | 19375 |
| 17 | 18 | 975 | 11875 |
| 18 | 12 | 675 | 11125 |
| 19 | 15 | 1225 | 10375 |
| 20 | 13 | 1075 | 18625 |

**C. Behavior of fraction dimensionality over time**

**Table SP3.** Average fraction dimensionality per simulation time step

| **Time-step** | **Fraction Dimensionality** |
| --- | --- |
| 0 | 0.0000 |
| 1 | 0.6601 |
| 2 | 0.9740 |
| 3 | 1.1570 |
| 4 | 1.2816 |
| 5 | 1.3703 |
| 6 | 1.4397 |
| 7 | 1.4951 |
| 8 | 1.5401 |
| 9 | 1.5775 |
| 10 | 1.6092 |
| 11 | 1.6365 |
| 12 | 1.6600 |
| 13 | 1.6811 |
| 14 | 1.6994 |
| 15 | 1.7158 |
| 16 | 1.7305 |
| 17 | 1.7436 |
| 18 | 1.7554 |
| 19 | 1.7661 |
| 20 | 1.7758 |
| 25 | 1.8136 |
| 30 | 1.8387 |
| 35 | 1.8563 |
| 40 | 1.8687 |
| 50 | 1.8844 |
| 60 | 1.8938 |
| 70 | 1.8999 |
| 80 | 1.9042 |
| 90 | 1.9073 |
| 100 | 1.9098 |
| 150 | 1.9168 |
| 200 | 1.9203 |

**D. Considerations of a generalized dimensionality measure**

Although the simulation assumes a constant value of $m$ for each possible attribute, scale elements do not necessarily need to be constrained to this. Having the same scale element allows to build regular spaces with equal axis length. This is done for the sake of convenience. However, below, we show with a numerical example that such an approximation does not harm the use and interpretation of the fraction dimension measure.

We offer next an extension of the fraction dimensionality with unequal number of scale elements per dimension.

If the *k*-dimensional frame space has $m_{i}$ scale elements along the *k-th* dimension, then the total number of unit cells in the frame space, $V_{o}$, is:

$V_{o}=\prod_{i=1}^{k} m_{i}$ (SP14)

Therefore, if there is a number $V$ of active cells in the space, the *generalized fraction dimensionality* can be defined as:

$gDIM=\frac{\ln V}{\ln\sqrt[k]{V_{o}}}=\frac{\ln V}{\ln\sqrt[k]{\prod_{i=1}^{k} m_{i}}}$ (SP15)

Equation (SP15) can be transformed as:

$gDIM=\frac{\ln V}{\ln\sqrt[k]{\prod_{i=1}^{k} m_{i}}}=\frac{\ln V}{\frac{1}{k}\sum_{i=1}^{k} ln(m_{i})}=\frac{k\ln V}{\sum_{i=1}^{k} ln(m_{i})}$ (SP16)

The generalized fraction dimensionality value in Eq. (SP16) is identical to Eq. (1) in the paper, whenever $m_{i}=m$ for all $i$:

$gDIM=\frac{k\ln V}{\sum_{i=1}^{k} \ln m}=\frac{k\ln V}{k\ln m}=\frac{\ln V}{\ln m}=DIM$ (SP17)

The generalized formula also nicely captures the saturation of the space. If considering a $k$-dimensional space with $m_{1}$ and $m_{2}$, the saturation is reached when all the cells are active, that is:

$gDIM=\frac{kln(m_{1}\cdot m_{2})}{ln(m_{1})+ln(m_{2})}=k$ (SP18)

Nonetheless, there are two apparent limitations of the $gDIM$ formula. First, it cannot precisely deliver intermediate integer dimensions. In a two-dimensional case, for instance, if one of the attributes is fully active while the other is inactive, the measure should deliver a value of 1. Consider the following example in a two-dimensional space with $m_{1}=10$ and $m_{2}=7$. On the one hand, if the first attribute is fully active, while the second is inactive, the generalized calculation results in $gDIM=\frac{2ln(10)}{ln(10)+\ln\left( 7 \right)}=1.03$. On the other hand, if the second is the fully active one, $gDIM=\frac{2ln(7)}{ln(10)+ln(7)}=0.87.$ Second, notice that $gDIM$ depends on $k$, unlike $DIM$. A calculation of $DIM$ will always give the same value, no matter what space size we are assuming.

A standing question is whether the difference between $DIM$ and $gDIM$ is absolutely relevant in capturing space dimensionality. In particular, it would important to assess whether the different $m_{i}$ values of the scale segments can be approximated to one single value (as in the agent-based model of the paper). Another important exploration is to assess how close a measure of $gDIM$ with a given $k$ value can capture $gDIM$ values with a lower $k$ (that is, to what extent values of ${gDIM}_{k'}$ capture the behavior of ${gDIM}_{k''}$, when $k^{'}>k''$).

The following example may clarify all of the above. Consider a *k*-dimensional space with $m_{1},m_{2},\ldots,m_{k}$ respective product variant values along the *k* axes. Let us assume three different spaces with $k$ values of 2, 5, and 10, where the larger space encapsulates the mid-size space, which in turn encapsulates the smaller one. Each dimension has a scale element drawn from a uniform distribution between 5 and 10, $m_{i}\sim U[5,10]$. We also build a fourth space, a hypothetical one where we approximate scale elements to one single averaged value. That is, we compute $DIM=\frac{\ln V}{\ln\overline{m}}$, where $\overline{m}=\mathrm{round}(\frac{\sum_{i=1}^{k} m_{i}}{k})$, and $k=10$.

Running several examples with different values reveals that all measures are very close to one another, and do not compromise the use and interpretation of the proposed dimensionality measure. Although $gDIM$ may capture dimensionality with different scale element values with some limitations, a reasonable approach is to use the $DIM$ formula with a single, averaged scale element value. See a numerical example in Figure SP1.

**
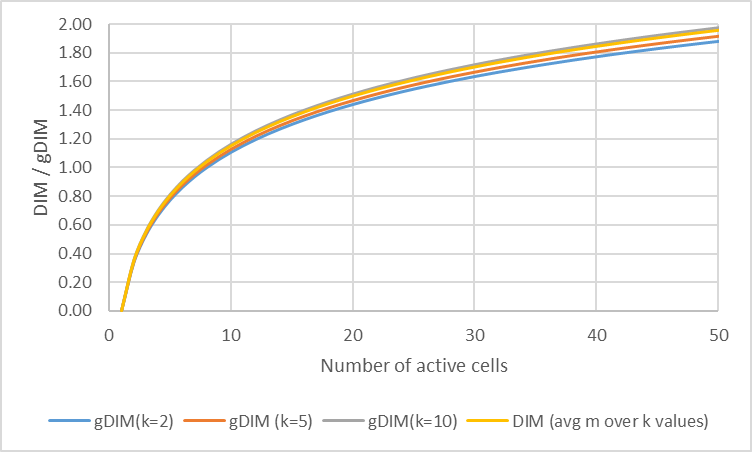
**

**Figure SP1.** Comparison of different fraction dimensionality measures
