# Supplementary material for: The coevolution of the firm and the product attribute space
Source: PLoS One. 2020 Jun 12;15(6):e0234007. doi: 10.1371/journal.pone.0234007 (PMC7292365; doi:10.1371/journal.pone.0234007)
Supplement: Attachment — Submitted filename: Review Report of PONE-D-19-34089 in PLOS ONE.doc [file pone.0234007.s002.doc]

Review Report of ***The Coevolution of the Firm and the Product Attribute Space*** by César García-Díaz, Gábor Péli, and Arjen van Witteloostuijn, which is considered to be published by ***PLOS ONE***

Under the framework of product attribute space, the competition among firms is studied in this paper. The authors first introduce the concept of fractional dimension to describe attribute space. And then they build their own models based on the traditional Cournot model and the networked Cournot model, respectively. At last, the numerical simulations are carried out by using the built computational models, to enclose the firms’ performances when new product variants are introduced. The model in this article is original and the research results are quite interesting. However, I still have several comments about this research, which are given as follows,

1. The section of Introduction is not complete. Obviously, the networked Cournot model is an important part of this paper. However, I cannot find any description of the networked Cournot model. Of course, no relevant literature in this field can be found. And I think the following two articles about R&D networks may be useful for the authors, which are given as,

[1] Gian Italo Bischi, Fabio Lamantia,A dynamic model of oligopoly with R&D externalities along networks. Part I., *Mathematics and Computers in Simulation*,Volume 84,2012,51-65.

[2] Gian Italo Bischi, Fabio Lamantia,A dynamic model of oligopoly with R&D externalities along networks. Part II., *Mathematics and Computers in Simulation*,Volume 84,2012,66-82.

[3] Herbert Dawid, Tim Hellmann,The evolution of R&D networks, *Journal of Economic Behavior & Organization*,Volume 105,2014, 158-172.

2. I can’t see any description of Figure 1 and Figure 2 from the paper. In fact, many authors often ignore the detail descriptions of figures, they obviously think that the readers can understand the figures. In fact, you need to add not only the descriptions of these figures, but also the conclusions you can draw from the figures.
3. The definition of fractal dimension is no longer fresh, but it is rare to use the fractal dimension to describe the degree of product differentiation. However, if this is not the first time to use fractal dimension to describe the degree of product differentiation, the authors need to add some references.
4. When the proper noun “cell” first appear in Line 119, the authors should give an explanation.
5. In Line 159, when talking about the fractal dimension, the authors assume a $k$ integer dimensional Euclidean space segment with $m$ scale elements per axis. As the authors said, each attribute of a commodity has multiple possible values, but the number of each scale element is always different, so we have $m_{i}\left(i=1,2,\cdots,k\right)$ and $m_{i}\neq m_{j}$. However, the authors assume $m_{1}=m_{2}=\cdots=m_{k}=m$ in the article without any interpretation. Another question is how to calculate the fractal dimension, when $m_{i}\neq m_{j}$.
6. When talking about the Cournot model, the following references may be useful.

[1] Y.H.Zhang, W.Zhou, T.Chu , Y.D.Chu, J.N.Yu, “Complex dynamics analysis for a two-stage Cournot duopoly game of semi-collusion in production,” *Nonlinear Dynamics*, vol. 91, no. 2, pp. 819- 835, 2018.

[2] J.Zhou, W.Zhou, T.Chu, Y.X.Chang, M.J.Huang, “Bifurcation, intermittent chaos and multi-stability in a two-stage Cournot game with R&D spillover and product differentiation,” *Applied Mathematics and Computation*, vol.341, pp.358-378, 2019.

# [3] [Y.X.Cao](https://www.worldscientific.com/doi/10.1142/S0218127419300313), [W.Zhou](https://www.worldscientific.com/doi/10.1142/S0218127419300313), [T.Chu](https://www.worldscientific.com/doi/10.1142/S0218127419300313), [Y.X.Chang](https://www.worldscientific.com/doi/10.1142/S0218127419300313), “Global Dynamics and Synchronization in a Duopoly Game with Bounded Rationality and Consumer Surplus,” *International Journal of bifurcation and chaos*, vol.29, no.11, Article ID: 1930031,2019

# [4] W.Zhou, X.X.Wang, "On the Stability and Multistability in a Duopoly Game with R&D Spillover and Price Competition," *Discrete Dynamics in Nature and Society*, vol. 2019, Article ID:2369898, 2019.

7. Form Line 215 to Line 220, the authors used a lot of symbols, such as $q_{i}$, $\pi_{A}$ and $q_{i}^{*}$ etc., without any interpretation.
8. From Line 223 to Line 232, I feel the authors' writing is a bit confusing and hard to understand.
9. In Fig.3, the authors assume that there are only two firms in the market and $m=11$, but I cannot see this expression in the paper. In addition, the calculation of Fig.3b is wrong. The authors said $DIM=1.72$ in the caption of Fig.3, and $DIM=1.8478$ in Fig.3b. However, 37 active cells can be found from Fig.3b, so the result should be $DIM=\frac{\ln37}{\ln121}\approx1.5059$.
10. In Line 436, the formula should be $DIM=1.78$, not $DIM=1\text{，}78$. Still in Line 436, $t=20$ is not accurate.
11. About the Fig.7a and Fig.7b, the authors should give a more detailed explanation. Please give me the reason why the authors use solid line between $f_{2}$ and $m_{3}$, as well as between $f_{3}$ and $m_{3}$. Why is there no connection between $f_{2}$ and $m_{4}$?
12. From Line 453 to Line 466, the authors should rewrite passage. I cannot understand the meaning of “5-6 introductions” and “time-step 4-5”.
13. In addition, there are also some language problems and spelling mistakes in this paper.
