# Supplementary material for: The coevolution of the firm and the product attribute space
Source: PLoS One. 2020 Jun 12;15(6):e0234007. doi: 10.1371/journal.pone.0234007 (PMC7292365; doi:10.1371/journal.pone.0234007)
Supplement: Attachment — Submitted filename: Response_to_referees_April_24_2020.docx [file pone.0234007.s003.docx]

**Response to referees**

***The Coevolution of the Firm and the Product Attribute Space***

**César García-Díaz, Gábor Péli, and Arjen van Witteloostuijn**

**Submitted to PLOS ONE**

We thank the reviewers for their very insightful comments on our work. Below, please find detail answers to each of their concerns.

***Reviewer #1***

*The paper develops an alternative way to analyze firms competition which differs from the traditional setup in which the dimensionality of the product attribute space is given. The focus of the analysis is to make the product attribute space endogenous depending on the firms' actions which, in turn, influence the firms' behavior. The analysis is conducted within a networked Cournot competition framework and a key role is played by the fraction dimensionality which reflects the saturation of the space with product varieties. The findings of the analysis reveal that, even a larger number of new products is introduced by firms with scale economies, the performance gap with firms that do not benefit from scale economies reduces as the fraction dimensionality increases. Overall the paper is pretty clear and well written and deals with an interesting topic related to firms competition, which is a relevant subject in the industrial sector analysis, and it may deserve publication in Plos One.*

Thank you very much.

*The authors may take into account the comments below:*

*1. In equation 3, I think a subscript i is missing after pi. Then authors may want to explain why they select a particular value of 0.2 for the mobility rate and those values for the demand parameters: is it only for an explanatory purpose? The conclusion at the end of sec. 3.1 depends on the parameter choice: is it possible to generalize it and find a condition under which profits are positive and the profit of scenario B is higher than A?*

Thanks for noting this. The subscript *i* to $\pi$ has been added, so equation (3) becomes:

$$\pi_{i}=\left( a-bQ \right)q_{i}-C\left( q_{i} \right), i=1,2$$

The mobility rate value of 0.2 is just an example. It is only for explanatory purposes. Notice that not only the mobility rate has a given value, but also the other parameters. Moreover, note that the example only considers two firms and two product variants. Our intention here is to show how the basic decision mechanism of the agent-based simulation operates, not to offer a general result.

This is the basic decision logic that firms use in the simulation model. Note that in the agent-based computational model there is no fixed mobility rate, but rather a distribution mechanism of demand that depends on the number of product variants.

The conclusions at the end of Section 3.1, of course, depend on the parameter choice, but this is only done to offer a numerical example of the basic ideas of the simulation. It would be possible to find conditions under which profits of scenario B are higher, but this is not the purpose of the example, given than the real model (the agent-based simulation) contains a more complex setting: a population of firms with different niche widths deciding on producing several product variants whose locations are also populated by other firms. Offering general conditions of the small example with only two firms would mislead the reader.

In order to clarify that this is only an example illustrating the basic dynamic of the simulation model, we have added an explanatory text at the beginning of Section 3.1.

*What are product coordinate values in fig.7 and what do they represent?*

Coordinates represent the different attributes of products. A product may be characterized by a number of attributes whose values define its location in attribute space. Attributes may be, e.g., degrees of alcohol and brand status (in beer), size, design functionalities, texture, colors, in home furniture, *et cetera*. Different combinations of attribute values would produce *qualitatively* different product variants. Note that this is different from considering attribute qualities in the model. Think about smartphones. Many may share the same number of attributes, but they may come in different qualities: high or low camera resolution, different sensitiveness of face recognition, and so forth. We are not considering attribute qualities. Neither are we considering demand features that allow different buying capacities for acquiring certain variants. They are out of the scope of the current paper, as they would lead to a whole set of different issues.

In summary, axes represent the different product attributes. As a unique response to both reviewers, we have modified the caption of Figure 7: “***An example of a competition network mapped onto a set of product variants.*** *Solid lines [panel (a)] are mappings from firms to products. Each firm serves a subset of the available products* $\{m_{1},m_{2},m_{3},m_{4}\}$*. Product nodes are embedded into a k-space (Freeman, 1983), with* $x_{i}$ *and* $y_{i}$ *denoting product coordinate values. Axes [panel (b)] represent values of product attributes. Dashed lines [panel (b)] indicate that two products are only differentiated in one of the attributes. Products* $m_{1}$ *and* $m_{3}$ *are horizontally differentiated along their x attribute; the same holds for* $m_{2}$ *and* $m_{4}$*.*”

We have also added the following lines before Figure 6: “*Figure 6 illustrates an example of an evolving bipartite network. Figure 7a shows a possible mapping between firms and product variants. Solid lines indicate links between firms and variants. Axes in Figure 7b represent values of product attributes. In this two-dimensional example, a given combination (x, y) of product attributes corresponds to a given product variant in attribute space. Solid lines indicate links between firms and variants. Firms open new variants that are minimally differentiated (they coincide with one of the attributes, but differentiate with the other). Dashed lines in Figure 7b indicate how variants are interrelated in attribute space.*”

*2. Figure 9 is not easy to read: maybe the reader would gain more insight from it if the fraction dimension was put on the vertical axis and the description of the figure at pag. 19 was enlarged by adding more economic intuition.*

The fraction dimensionality on the vertical axis is displayed in Figure 8. For the sake of clarity, we have modified Figure 9 in order to only show the evolution of the number of product variants over time. The economic intuition is that firms find it profitable to open new variants at the onset of the market; the aggregated actions trigger an explosion of variant generation per time-step that peak at around 5.5 variants, on average. These aggregated actions of firms spread out the demand across the attribute space, implying that further incentives to open new variants sharply decline over time. Firms face the tradeoff between further expansion and niche width costs; expansion in a saturated market is discouraged because of the difficulties to capture incremental demand.

The new Figure 9 is the following:

This economic intuition has been added to the caption of Figure 9, as follows: “*Firms find it profitable to open new variants at the onset of the market; the aggregated actions trigger an explosion of variant creation per time-step that peaks at around 5.5 variants, on average. These aggregated actions of firms spread out the demand across the attribute space, implying that further incentives to open new variants sharply decline over time.*”

*3. The description of fig. 10 should also be improved when explaining the concentration issue.*

This explanation is already in place on pages 20-21 of the manuscript. In order to further complement the explanation, we have added the following short caption to Figure 10: “*Market concentration first declines as a consequence of variant opening, which leads to firm distribution across the space and relief of direct competition. When the market gradually approaches saturation, variant opening declines and direct competition rises, favoring those with enough scale advantage to increase their market shares. Although qualitatively expected in terms of initial decline and further increase, market concentration values remain low due to model design decisions of only two firm types and a constant firm population.”*

***Reviewer #2***

*Under the framework of product attribute space, the competition among firms is studied in this paper. The authors first introduce the concept of fractional dimension to describe attribute space. And then they build their own models based on the traditional Cournot model and the networked Cournot model, respectively. At last, the numerical simulations are carried out by using the built computational models, to enclose the firms’ performances when new product variants are introduced. The model in this article is original and the research results are quite interesting. However, I still have several comments about this research, which can be found in the appendix.*

*Appendix:*

Review Report of ***The Coevolution of the Firm and the Product Attribute Space*** by César García-Díaz, Gábor Péli, and Arjen van Witteloostuijn, which is considered to be published by ***PLOS ONE***

Under the framework of product attribute space, the competition among firms is studied in this paper. The authors first introduce the concept of fractional dimension to describe attribute space. And then they build their own models based on the traditional Cournot model and the networked Cournot model, respectively. At last, the numerical simulations are carried out by using the built computational models, to enclose the firms’ performances when new product variants are introduced. The model in this article is original and the research results are quite interesting. However, I still have several comments about this research, which are given as follows,

1. The section of Introduction is not complete. Obviously, the networked Cournot model is an important part of this paper. However, I cannot find any description of the networked Cournot model. Of course, no relevant literature in this field can be found. And I think the following two articles about R&D networks may be useful for the authors, which are given as,

[1] Gian Italo Bischi, Fabio Lamantia,A dynamic model of oligopoly with R&D externalities along networks. Part I., *Mathematics and Computers in Simulation*,Volume 84,2012,51-65.

[2] Gian Italo Bischi, Fabio Lamantia,A dynamic model of oligopoly with R&D externalities along networks. Part II., *Mathematics and Computers in Simulation*,Volume 84,2012,66-82.

[3] Herbert Dawid, Tim Hellmann,The evolution of R&D networks, *Journal of Economic Behavior & Organization*,Volume 105,2014, 158-172.

The networked Cournot model is not really a model. Is a particular setting where competition *à la* Cournot takes place between networked players. Recent studies illustrating Cournot competition in a networked setting are already cited in the manuscript. The papers of Abolhassani et al. (2014) and Bimpikis et al. (2019) illustrate game-theoretical approaches to competition in bipartite networks of firms and markets. The work of Bischi and Lamantia (2012, 2012b), and Dawid and Hellmann (2014) are important antecedents. Nonetheless, they differ from Abolhassani et al. (2014), Bimpikis et al. (2019), and our work in the way network structures affect Cournot competition outcomes. In Bischi and Lamantia (2012, 2012b) and Dawid and Hellmann (2014), firms first establish R&D networks that affect their marginal cost of production, and then compete in a market by calculating optimal quantity outcomes. Our work portraits an evolving bipartite network of firms and product variants.

Part of the above text and the suggested references have been added to the manuscript (see page 11).

References

Abolhassani M, Bateni MH, Hajiaghayi M, Mahini H, Sawant A. Network cournot competition. Proceedings of the 10th International Conference on Web and Internet Economics, WINE 2014, December 14-17, Beijing, China. Cham: Springer; 2014.

Bimpikis K, Ehsani S, İlkılıç R. Cournot competition in networked markets. Management Science. 2019 Feb 19;65(6):2467-81.

2. I can’t see any description of Figure 1 and Figure 2 from the paper. In fact, many authors often ignore the detail descriptions of figures, they obviously think that the readers can understand the figures. In fact, you need to add not only the descriptions of these figures, but also the conclusions you can draw from the figures.

We have modified / extended the current caption of the figures, as follows:

Caption of Figure 1: “***Example of strategic alternatives for new competitor’s positioning.*** *A space with two existing product attributes (a and b) and two established firms (firms 1 and 2) is depicted. The figure exemplifies three different strategic positioning alternatives for a new competitor: (i), (ii), and (iii). A new market rival could opt for: (i) competing at locations where firms are already established (dashed lines indicate Firm 1 and Firm 2’s niches), (ii) occupying existing but empty spaces, or (iii) developing new product attributes (e.g., attribute c). This figure is our own elaboration, but inspired by that of Ofek (2008).*”

Caption of Figure 2: “***Increasing perceived usefulness for consumers by adding features (attributes) to products.*** *Attributes add value to consumers at an increasing rate up to a certain point. Further additions add value at a decreasing rate. Beyond a certain number of features, additional ones might not add any value. This figure is our own elaboration, but inspired by that of Warren (2008).*”

Conclusions drawn from the figures were already in the manuscript. These figures illustrate the basic motivations of the paper. On the one hand, firms can open new attributes as a result of a strategic decision [in contrast with the alternatives of invading existing variants or serving existing but unused spot of the space (see page 3, lines 77-79 in the manuscript for a reference to Figure 1)]. On the other hand, generating new attributes by creating new product features has been long part of the strategic moves of firms because of the increased attractiveness to consumers (see page 4, lines 107-110 in the manuscript for a reference to Figure 2).

3. The definition of fractal dimension is no longer fresh, but it is rare to use the fractal dimension to describe the degree of product differentiation. However, if this is not the first time to use fractal dimension to describe the degree of product differentiation, the authors need to add some references.

Although we acknowledge that fractal dimensionality has been used to measure properties of financial time series, we have not found any paper that uses similar concepts to measure structural properties of the product attribute space, with the exception of a paper already cited in the manuscript (García-Díaz et al., 2008). In that paper, the fractal dimensionality notion is used with a slightly different purpose: the inspection of the progression of profit/cost ratios of large-scale and small-scale firms in an evolving entry-and-exit market.

However, we did find a few papers that have used fractal dimensionality to explore properties of quantity and price time series: a dynamic Cournot competition with differentiated goods (Fanti and Gori, 2012), and a duopoly Bertrand competition model in Yi and Zheng (2015).

These references have been added to the manuscript (see footnote 2).

Added references:

Fanti, L., & Gori, L. (2012). The dynamics of a differentiated duopoly with quantity competition. *Economic Modelling*, 29(2), 421-427.

Yi, Q. G., & Zeng, X. J. (2015). Complex dynamics and chaos control of duopoly Bertrand model in Chinese air-conditioning market. *Chaos, Solitons & Fractals*, 76, 231-237.

4. When the proper noun “cell” first appear in Line 119, the authors should give an explanation.

We have deleted the word “cell” in that line. The term is better understood later in the manuscript when referring to Figure 3 and the calculation of fraction dimensionality (Page 6).

5. In Line 159, when talking about the fractal dimension, the authors assume a $k$ integer dimensional Euclidean space segment with $m$ scale elements per axis. As the authors said, each attribute of a commodity has multiple possible values, but the number of each scale element is always different, so we have $m_{i}\left(i=1,2,\cdots,k\right)$ and $m_{i}\neq m_{j}$. However, the authors assume $m_{1}=m_{2}=\cdots=m_{k}=m$ in the article without any interpretation. Another question is how to calculate the fractal dimension, when $m_{i}\neq m_{j}$.

The following text has been added to the supplementary material (S1 File):

Although the simulation assumes a constant value of $m$ for each possible attribute, scale elements do not necessarily need to be constrained to this. Having the same scale element allows to build regular spaces with equal axis length. This is done for the sake of convenience. However, below, we show with a numerical example that such an approximation does not harm the use and interpretation of the fraction dimension measure.

We offer next an extension of the fraction dimensionality with unequal number of scale elements per dimension.

If the *k*-dimensional frame space has $m_{i}$ scale elements along the *k-th* dimension, then the total number of unit cells in the frame space, $V_{o}$, is:

$V_{o}=\prod_{i=1}^{k} m_{i}$	(SP14)

Therefore, if there is a number $V$ of active cells in the space, the *generalized fraction dimensionality* can be defined as:

$gDIM=\frac{\ln V}{\ln\sqrt[k]{V_{o}}}=\frac{\ln V}{\ln\sqrt[k]{\prod_{i=1}^{k} m_{i}}}$	(SP15)

Equation (SP15) can be transformed as:

$gDIM=\frac{\ln V}{\ln\sqrt[k]{\prod_{i=1}^{k} m_{i}}}=\frac{\ln V}{\frac{1}{k}\sum_{i=1}^{k} ln(m_{i})}=\frac{k\ln V}{\sum_{i=1}^{k} ln(m_{i})}$	(SP16)

The generalized fraction dimensionality value in Eq. (SP16) is identical to Eq. (1) in the paper, whenever $m_{i}=m$ for all $i$:

$gDIM=\frac{k\ln V}{\sum_{i=1}^{k} \ln m}=\frac{k\ln V}{k\ln m}=\frac{\ln V}{\ln m}=DIM$	(SP17)

The generalized formula also nicely captures the saturation of the space. If considering a $k$-dimensional space with $m_{1}$ and $m_{2}$, the saturation is reached when all the cells are active, that is:

$gDIM=\frac{kln(m_{1}\cdot m_{2})}{ln(m_{1})+ln(m_{2})}=k$	(SP18)

Nonetheless, there are two apparent limitations of the $gDIM$ formula. First, it cannot precisely deliver intermediate integer dimensions. In a two-dimensional case, for instance, if one of the attributes is fully active while the other is inactive, the measure should deliver a value of 1. Consider the following example in a two-dimensional space with $m_{1}=10$ and $m_{2}=7$. On the one hand, if the first attribute is fully active, while the second is inactive, the generalized calculation results in $gDIM=\frac{2ln(10)}{ln(10)+\ln\left( 7 \right)}=1.03$. On the other hand, if the second is the fully active one, $gDIM=\frac{2ln(7)}{ln(10)+ln(7)}=0.87.$ Second, notice that $gDIM$ depends on $k$, unlike $DIM$. A calculation of $DIM$ will always give the same value, no matter what space size we are assuming.

A standing question is whether the difference between $DIM$ and $gDIM$ is absolutely relevant in capturing space dimensionality. In particular, it would important to assess whether the different $m_{i}$ values of the scale segments can be approximated to one single value (as in the agent-based model of the paper). Another important exploration is to assess how close a measure of $gDIM$ with a given $k$ value can capture $gDIM$ values with a lower $k$ (that is, to what extent values of ${gDIM}_{k'}$ capture the behavior of ${gDIM}_{k''}$, when $k^{'}>k''$).

The following example may clarify all of the above. Consider a *k*-dimensional space with $m_{1},m_{2},\ldots,m_{k}$ respective product variant values along the *k* axes. Let us assume three different spaces with $k$ values of 2, 5, and 10, where the larger space encapsulates the mid-size space, which in turn encapsulates the smaller one. Each dimension has a scale element drawn from a uniform distribution between 5 and 10, $m_{i}\sim U[5,10]$. We also build a fourth space, a hypothetical one where we approximate scale elements to one single averaged value. That is, we compute $DIM=\frac{\ln V}{\ln\overline{m}}$, where $\overline{m}=\mathrm{round}(\frac{\sum_{i=1}^{k} m_{i}}{k})$, and $k=10$.

Running several examples with different values reveals that all measures are very close to one another, and do not compromise the use and interpretation of the proposed dimensionality measure. Although $gDIM$ may capture dimensionality with different scale element values with some limitations, a reasonable approach is to use the $DIM$ formula with a single, averaged scale element value. See a numerical example in Figure SP1.

**Figure SP1.** Comparison of different fraction dimensionality measures

6. When talking about the Cournot model, the following references may be useful.

[1] Y.H.Zhang, W.Zhou, T.Chu , Y.D.Chu, J.N.Yu, “Complex dynamics analysis for a two-stage Cournot duopoly game of semi-collusion in production,” *Nonlinear Dynamics*, vol. 91, no. 2, pp. 819- 835, 2018.

[2] J.Zhou, W.Zhou, T.Chu, Y.X.Chang, M.J.Huang, “Bifurcation, intermittent chaos and multi-stability in a two-stage Cournot game with R&D spillover and product differentiation,” *Applied Mathematics and Computation*, vol.341, pp.358-378, 2019.

# [3] [Y.X.Cao](https://www.worldscientific.com/doi/10.1142/S0218127419300313), [W.Zhou](https://www.worldscientific.com/doi/10.1142/S0218127419300313), [T.Chu](https://www.worldscientific.com/doi/10.1142/S0218127419300313), [Y.X.Chang](https://www.worldscientific.com/doi/10.1142/S0218127419300313), “Global Dynamics and Synchronization in a Duopoly Game with Bounded Rationality and Consumer Surplus,” *International Journal of bifurcation and chaos*, vol.29, no.11, Article ID: 1930031,2019

# [4] W.Zhou, X.X.Wang, "On the Stability and Multistability in a Duopoly Game with R&D Spillover and Price Competition," *Discrete Dynamics in Nature and Society*, vol. 2019, Article ID:2369898, 2019.

These references have been added to the manuscript and cited in the paragraph where we discuss Cournot competition models (page 8).

7. Form Line 215 to Line 220, the authors used a lot of symbols, such as $q_{i}$, $\pi_{A}$ and $q_{i}^{*}$ etc., without any interpretation.

We have fixed this in the document: $q_{i}$is the quantity produced by firm *i*, $\pi_{A,i}$ is the profit firm *i* get under scenario *A*, and $q_{i}^{*}$is firm *i*’s optimal quantity.

8. From Line 223 to Line 232, I feel the authors' writing is a bit confusing and hard to understand.

These lines are part of the numerical example we offer to explain the basic workings of the model. What we are saying is that, in the case a firm opens a new variant, it has to compute how many consumers will gain from this, in comparison with the number of consumers it will get under competition in the existing variant. We have re-written these lines as follows:

“*Assume that the cost of opening a new variant is K, and the cost of handling the complexity of more than one variant is N. The N cost accounts for the scope diseconomies firms incur when serving demand from several markets (niche spanning costs; see García-Díaz et al., 2008). For the sake of demonstration in the example, we set K = N = 1.*

*Consumers may be lured to consume the new variant. For that, we assume that consumers are willing to switch to a new variant with a mobility rate* $\vartheta=0.2$*. That is, 20% would be willing to consume the new variant. Notice that if* $a/b$ *is aggregated demand, and if the intercept value* $a$ *is kept constant, the new variant has a potential demand of* $\vartheta(\frac{a}{b})=a/(\frac{b}{\vartheta})$*, so the slope of the price equation for the second variant would be* $b/\vartheta$ *(that is, the market* $\vartheta(\frac{a}{b})$ *clears when the price equals zero).^[1]^ A similar change happens to the existing variant, with its demand becoming* $(1-\vartheta)(\frac{a}{b})$*.*

*Knowing this, Firm 2 would open a new variant if its monopoly profit in the new market will offset the sum of investment K, the additional cost N, and the profit loss that results from redistributing the market between two product variants instead of one (see scenario B in Fig 5).*”

9. In Fig.3, the authors assume that there are only two firms in the market and $m=11$, but I cannot see this expression in the paper. In addition, the calculation of Fig.3b is wrong. The authors said $DIM=1.72$ in the caption of Fig.3, and $DIM=1.8478$ in Fig.3b. However, 37 active cells can be found from Fig.3b, so the result should be $DIM=\frac{\ln37}{\ln121}\approx1.5059$.

In the full model, the market has *F* number of firms (Section 3.2). Only the expository example has two firms. The example with two firms is only presented in order to show the basic workings of the model. Indeed, the value of *m* is arbitrary and we have assumed throughout the paper that, without loss of generality, it can take values from 0 to 10 (that is, 11 possible values). We have added the corresponding modification on page 19 to clarify this.

The reviewer is right about the miscalculation of the dimensionality value. However, we clarify that the correct formula is $DIM=\frac{\ln V}{\ln m}$ (the denominator is 11, not 121). We were using an incorrect figure / label for Figure 3b. In the following example, $DIM=\frac{\ln62}{\ln11}=1.72$. This matches the below figure, which has replaced the old Figure 3b:

10. In Line 436, the formula should be $DIM=1.78$, not $DIM=1\text{，}78$. Still in Line 436, $t=20$ is not accurate.

The typo has been corrected. The mention of $t=20$ is not accurate, and actually is wrong. Thank you for noting this. In fact, the near-stability is slowly approached after $t=40$ (the numbers are in Table SP3), when *DIM* ≈ 1.87. This has been corrected in the manuscript.

11. About the Fig.7a and Fig.7b, the authors should give a more detailed explanation. Please give me the reason why the authors use solid line between $f_{2}$ and $m_{3}$, as well as between $f_{3}$ and $m_{3}$. Why is there no connection between $f_{2}$ and $m_{4}$?

These figures are an expository example. It presents a hypothetical instance to illustrate how firms form a bipartite network with products (variants). This figure is a building block of what we later mean with variants embedded in an *k*-dimensional space (see Figure 7b). In the example, firms do not need to be connected to all variants (exactly as it happens in the simulation). Every firm is connected to three out four possible variants. Solid lines indicate links between firms and variants. Since firms open new variants that are minimally differentiated (they coincide in one of the attributes), dashed lines indicate how variants are interrelated in the attribute space.

We have modified the caption of Figure 7 as follows: “***An example of a competition network mapped onto a set of product variants.*** *Solid lines [panel (a)] are mappings from firms to products. Each firm serves a subset of the available products* $\{m_{1},m_{2},m_{3},m_{4}\}$*. Product nodes are embedded into a k-space (Freeman, 1983), with* $x_{i}$ *and* $y_{i}$ *denoting product coordinate values. Axes [panel (b)] represent values of product attributes. Dashed lines [panel (b)] indicate that two products are only differentiated in one of the attributes. Products* $m_{1}$ *and* $m_{3}$ *are horizontally differentiated along their x attribute; the same holds for* $m_{2}$ *and* $m_{4}$*.*”

This preceding paragraph has also been added, before Figure 6: “*Figure 6 illustrates an example of an evolving bipartite network. Figure 7a shows a possible mapping between firms and product variants. Solid lines indicate links between firms and variants.* *Axes in Figure 7b represent values of product attributes. In this two-dimensional example, a given combination (x, y) of product attributes correspond to a given product variant in attribute space. Solid lines indicate links between firms and variants. Firms open new variants that are minimally differentiated (they coincide with one of the attributes, but differentiate with the other). Dashed lines in Figure 7b indicate how variants are interrelated in attribute space.*”

12. From Line 453 to Line 466, the authors should rewrite passage. I cannot understand the meaning of “5-6 introductions” and “time-step 4-5”.

We have rewritten the paragraph as follows:

“*Figure 9 illustrates the frequency of product introduction along the simulation time span. Results reveal that the speed of new variant creation reaches a peak of about 5.5 variants per step quite early, at around time-step 5, when DIM≈1.37 (see average DIM values per simulation step in the supplementary material, S1 File). From that time-step on, variant creation drops drastically. Average new variant creation approximates zero way before reaching half simulation time. Dragging consumers to new product variants becomes less profitable, partly because the constant total demand thins out with the increasing number of product variants in place. Efficiency improvement through niche expansion becomes marginal; firms already securing an efficient producer status dominate at the detriment of the least efficient.”*

We hope it looks clearer now.

13. In addition, there are also some language problems and spelling mistakes in this paper.

We have performed a thorough revision of grammar and language style to ensure there are not spelling mistakes in this new version.

Important note:

In an email we received from the editorial office (i.e., Vicky Stabler, on 5 march 2020), we were supposedly required to provide copyrights of Figs 1 and 2, as if they were copied to our paper.

We clarified that the two figures are our elaboration but inspired in figures from the existing literature (see caption of Figs 1 and 2 in the manuscript). Vicky Stabler replied to us clarifying the misunderstanding: In her words (March 17, 2020): "If your figures were created by yourselves, then please could you add this information to your response to reviewer file and this will be fine to proceed."

We state in the figures' caption that the figures are our own elaboration, as well as in the submission form. For the editorial office information, the figures of reference are included in the manuscript data, along with the other manuscript files.

1. Of course, the price equation for the second variant may change in different ways (both the intercept *a* and the slope *b*). We assume that the intercept is fixed because: (i) to obtain positive quantities, *a* has to be set much larger than unit cost *c*, so a decrease of *a* for subsequent product variants is not desirable for modeling purposes; and (ii) an increase of *a* for subsequent product variants would likely generate higher prices, rendering the investigation of whether firms would open new variants trivial as new variants usually charge higher prices. Thus, a more restrictive way of exploring the model is, first, assuming *a* fixed while *b* is recalculated according to the demand allocated to the product variants and, second, assuming that the market is cleared when the price equals zero. We also adopt these assumptions in full the computational model.
